# Adverse Events Reported During Weekly Isoniazid-Rifapentine (3HP) Tuberculosis Preventive Treatment Among People With Human Immunodeficiency Virus in Uganda

**DOI:** 10.1093/ofid/ofae667

**Published:** 2024-11-14

**Authors:** Jillian L Kadota, Allan Musinguzi, Hélène E Aschmann, Lydia Akello, Fred Welishe, Jane Nakimuli, Christopher A Berger, Noah Kiwanuka, Patrick P J Phillips, Achilles Katamba, David W Dowdy, Adithya Cattamanchi, Fred C Semitala

**Affiliations:** Division of Pulmonary and Critical Care Medicine, University of California, San Francisco, San Francisco, California, USA; Center for Tuberculosis, University of California, San Francisco, San Francisco, California, USA; Department of Research, Infectious Diseases Research Collaboration, Kampala, Uganda; Division of Pulmonary and Critical Care Medicine, University of California, San Francisco, San Francisco, California, USA; Center for Tuberculosis, University of California, San Francisco, San Francisco, California, USA; Department of Research, Infectious Diseases Research Collaboration, Kampala, Uganda; Department of Research, Infectious Diseases Research Collaboration, Kampala, Uganda; Department of Research, Infectious Diseases Research Collaboration, Kampala, Uganda; Division of Pulmonary and Critical Care Medicine, University of California, San Francisco, San Francisco, California, USA; Center for Tuberculosis, University of California, San Francisco, San Francisco, California, USA; Department of Epidemiology and Biostatistics, School of Public Health, Makerere University College of Health Sciences, Kampala, Uganda; Division of Pulmonary and Critical Care Medicine, University of California, San Francisco, San Francisco, California, USA; Center for Tuberculosis, University of California, San Francisco, San Francisco, California, USA; Department of Medicine, Clinical Epidemiology and Biostatistics Unit, Makerere University College of Health Sciences, Kampala, Uganda; Uganda Tuberculosis Implementation Research Consortium, Walimu, Kampala, Uganda; Department of Epidemiology, Johns Hopkins Bloomberg School of Public Health, Baltimore, Maryland, USA; Center for Tuberculosis, University of California, San Francisco, San Francisco, California, USA; Division of Pulmonary Diseases and Critical Care Medicine, University of California, Irvine, Orange, California, USA; Department of Research, Infectious Diseases Research Collaboration, Kampala, Uganda; Department of Medicine, Makerere University College of Health Sciences, Kampala, Uganda; Department of Research, Makerere University Joint AIDS Program, Kampala, Uganda

**Keywords:** adverse events, effectiveness-implementation hybrid, HIV/AIDS, isoniazid-rifapentine, tuberculosis preventive therapy

## Abstract

**Background:**

Short-course tuberculosis (TB) prevention regimens, including 12 weeks of isoniazid and rifapentine (3HP), are increasingly used in high-TB-burden countries. Despite established safety and tolerability in efficacy trials, 3HP-related adverse events (AEs) could differ in routine settings. Real-world data on AE type, frequency, and timing are crucial for health systems considering 3HP programmatic scale-up.

**Methods:**

We reviewed AEs among people with human immunodeficiency virus (HIV) participating in a pragmatic implementation trial of facilitated 3HP taken by directly observed therapy (DOT) or self-administered therapy (SAT) in Kampala, Uganda, and classified them using the Common Terminology Criteria for Adverse Events. We assessed AE timing and summarized related clinical actions including laboratory tests, diagnoses made, medications prescribed, and treatment interruptions.

**Results:**

Among 1655 people with HIV treated between July 2020 and September 2022, 270 (16.3%) reported 451 events; main issues included general (7%), nervous system (6%), musculoskeletal (5%), gastrointestinal (5%), and dermatologic (3%) disorders. Most (61%) occurred within 6 weeks of initiating 3HP. Among those with events, 211 (78%) required further clinician evaluation, 202 (75%) required laboratory testing, 102 (38%) had medications prescribed, 40 (15%) had treatment paused, and 14 (5%) discontinued 3HP. Women, those multidimensionally impoverished, and DOT recipients were more likely to report an AE. SAT users and later enrollees were more likely to have 3HP interrupted or stopped due to an AE.

**Conclusions:**

In a routine setting, 3HP was safe, with 16% of people with HIV reporting AEs and only 3% requiring temporary or permanent treatment interruption. These findings support 3HP expansion in routine HIV/AIDS care settings for TB prevention.

**Clinical Trials Registration.** NCT03934931.

## Teaser

Weekly isoniazid and rifapentine is being rolled out for tuberculosis prevention in high-burden countries. We describe the incidence and clinical management of adverse events in a real-world clinical setting among people with HIV participating in a pragmatic implementation study in Uganda.

Prevention of tuberculosis (TB) is an urgent priority for global TB programs [1, 2], and scale-up of TB preventive treatment (TPT) in high-TB-burden countries is critical for achieving ambitious TB elimination goals by 2030. Short-course regimens, such as 12 weekly doses of isoniazid and rifapentine (3HP), are now recommended as an option for TB prevention based on several trials demonstrating efficacy, safety, and improved completion relative to the traditional standard of 6–9 months of daily isoniazid [3–6]. However, data on the safety of 3HP in routine care settings remain sparse and are needed to help policymakers better anticipate the resources required for adverse event (AE) management as 3HP is rolled out for TB prevention.

Here, we describe AEs reported in the 3HP Options Trial, a pragmatic implementation trial that delivered 3HP to adults with human immunodeficiency virus (HIV) accessing routine clinical HIV/AIDS care in Kampala, Uganda, via facilitated directly observed therapy (DOT), facilitated self-administered therapy (SAT), or participant choice between facilitated DOT and SAT (hereafter “Choice”). In this pragmatic trial, routine health workers were responsible for monitoring, evaluating, and providing care to participants receiving 3HP treatment. We previously reported that treatment completion was high overall (94%) and similar across trial arms (95%, 92%, and 94% for facilitated DOT, facilitated SAT, and Choice, respectively) [7]. Our objective here was to characterize the frequency, type, and severity of AEs, and secondarily to identify demographic and clinical characteristics associated with reported AEs and with 3HP treatment interruption or discontinuation.

## METHODS

### Study Design and Population

The study design and population are described in detail elsewhere [7, 8]. In brief, the 3HP Options Trial was a pragmatic trial of 3HP delivery strategies among people aged ≥18 years with HIV (PWH) accessing care at the Mulago Immune Suppression Syndrome (ISS; ie, HIV/AIDS) clinic, the largest HIV/AIDS clinic in Uganda. PWH were included in the study if they weighed at least 40 kg and could provide written informed consent in English or Luganda. PWH were excluded in case of presumptive or current TB, concomitant medications or antiretroviral therapy (ART) contraindicated with rifapentine, a history of sensitivity to isoniazid or rifamycins, contact with a patient with known drug-resistant TB, completion of TB treatment or isoniazid preventive therapy in the past 2 years, and documented clinical liver disease. Other exclusions included prisoners, PWH not intending to remain within 25 km of the Mulago ISS clinic, those without access to a mobile phone, household members of enrolled participants, and women who were pregnant or breastfeeding. Women who became pregnant during 3HP treatment would continue and be monitored in accordance with the Uganda National TPT Guidelines. Eligible PWH were randomized to 1 of 3 delivery strategies: facilitated DOT, facilitated SAT, or participant choice between facilitated DOT and SAT. After taking the first dose in person, participants taking 3HP via facilitated DOT returned to the clinic weekly for the remaining 11 doses, whereas those taking 3HP via facilitated SAT had scheduled clinic visits for in-person dosing at weeks 6 and 12 only.

### Adverse Event Reporting and Assessment

Prior to 3HP dosing at scheduled clinic visits, a Mulago ISS clinic pharmacy technician used a standardized form to screen participants for the presence/absence of any side effect and, if present, to screen for each of the following commonly reported side effects of 3HP treatment: loss of appetite; nausea or vomiting; yellow eyes or skin; abdominal pain; diarrhea; rash/hives; fever or chills; dizziness/fainting; numbness or tingling; headache; joint pain; itching; weakness; or other (with the option to specify). Following self-administered doses, participants taking 3HP by SAT could report that they were feeling unwell via weekly 2-way toll-free interactive voice response (IVR) phone calls sent by a digital adherence monitoring platform used for the study (99DOTS, Everwell Health Solutions, Bengaluru, India). Mulago ISS clinic pharmacy technicians made phone calls to participants who reported feeling unwell and screened for side effects using the same standardized form used during in-person visits. In addition, all participants were instructed that they could visit or call the clinic at any time during open hours if they felt unwell, without the need to wait for their next scheduled appointment. Thus, participants could report AEs at scheduled clinic visits for in-person dosing, IVR phone calls (SAT only), or through unscheduled phone calls and clinic visits.

Mulago ISS clinic pharmacy technicians recorded the presence or absence of each common side effect using the standardized form. The form also included fields to specify if the patient was referred for further evaluation by a clinician, if or which laboratory tests were ordered, results of any laboratory testing, medications prescribed because of AE(s) reported, and details about treatment interruption or permanent discontinuation. Of note, Mulago ISS clinic pharmacy technicians made all decisions to refer participants with suspected AEs to a routine (ie, nonstudy) clinician who then managed all activities related to AEs assessment, including additional clinical or laboratory evaluation and decisions to hold or discontinue 3HP treatment.

### Statistical Analysis

At the conclusion of participant follow-up, 2 analysts (J. L. K. and H. E. A.) independently recoded all AEs initially recorded as “other” (eg, those not in predefined categories and/or those with different spellings). The same analysts then independently recategorized all AEs using the Common Terminology Criteria for Adverse Events (CTCAE), version 5.0 [9]. Two research staff with medical training (A. M. and L. A.) adjudicated disagreements in categorizations.

Time to AE onset was calculated by taking the difference between the date of first report of the AE and the date of 3HP initiation. We quantified AEs that resulted in referrals, additional laboratory tests ordered, diagnoses made, and medications prescribed using counts and percentages, and compared across study arms using Fisher exact tests. Finally, we used multivariable logistic regression to explore the clinical and sociodemographic variables associated with reporting any AE and discontinuing treatment because of an AE. Variables included in final multivariate models were those determined a priori based on relationships identified in the literature [10, 11].

The trial is registered at ClinicalTrials.gov (NCT03934931) and was approved by the ethical committees at the University of California, San Francisco, the Makerere University College of Health Sciences School of Public Health, and the Uganda National Council for Science and Technology. All participants gave written informed consent for study participation.

## RESULTS

Between 13 July 2020 and 8 July 2022, we enrolled 1655 eligible PWH. Follow-up continued until 29 September 2022. In the Choice arm, 370 participants (67.0%) initially preferred facilitated DOT; thus, 921 participants received 3HP via facilitated DOT, and 734 participants via facilitated SAT. Median participant age was 42 years (interquartile range [IQR], 36–48 years); 1122 (67.8%) were female; and median time on ART was 9.0 years (IQR, 5.6–12.5 years), with no differences by treatment strategy (Table 1).

**Table 1.** Participant Characteristics Comparing Those Taking Isoniazid and Rifapentine Once Weekly for 12 Weeks via Directly Observed Therapy Versus Self-administered Therapy (N = 1655)

Characteristic	DOT (n = 921)	SAT (n = 734)
Age, y, mean (SD)	41.9 (9.4)	42.6 (9.2)
Female sex	614 (66.7)	508 (69.2)
Education		
None	71 (7.7)	68 (9.3)
Primary	452 (49.1)	330 (45.0)
Secondary	335 (36.4)	265 (36.1)
Tertiary/vocational	37 (4.0)	48 (6.5)
University/graduate school	26 (2.8)	23 (3.1)
Employment status^a^		
Unemployed	129 (14.0)	96 (13.1)
Hired worker	105 (11.4)	99 (13.5)
Self-employed worker	445 (48.3)	372 (50.7)
Temporary/informal worker	233 (25.3)	163 (22.2)
Other	3 (0.3)	2 (0.3)
Multidimensional Poverty Index^b^		
Not vulnerable to multidimensional poverty	378 (41.0)	341 (46.5)
Vulnerable to multidimensional poverty	332 (36.1)	251 (34.2)
Multidimensionally poor	167 (18.1)	118 (16.1)
Severely multidimensionally poor	44 (4.8)	24 (3.3)
Reported an AE while taking 3HP	175 (19.0)	95 (12.9)
Prior TB infection	165 (17.9)	136 (18.5)
Time on ART, y, mean (SD)	8.8 (4.5)	8.9 (4.4)
ART regimen		
Dolutegravir + lamivudine + tenofovir	804 (87.3)	647 (88.2)
Tenofovir + lamivudine + efavirenz	65 (7.1)	46 (6.3)
Other^c^	52 (5.6)	41 (5.6)
Viral load <1000 copies/mL^d^	906 (98.4)	727 (99.1)
Body mass index, kg/m^2^, mean (SD)	25.9 (5.8)	26.3 (5.4)

Data are presented as No. (%) unless otherwise indicated.

Abbreviations: 3HP, isoniazid-rifapentine once weekly for 12 weeks; AE, adverse event; ART, antiretroviral therapy; DOT, directly observed therapy; SAT, self-administered therapy; SD, standard deviation; TB, tuberculosis.

^a^n = 8 missing.

^b^The global Multidimensional Poverty Index (MPI) examines deprivations across 10 indicators in dimensions of health, education, and standards of living, with those deprived in one-third or more of the 10 indicators counted as being multidimensionally poor. MPI scores can range from 0 to 1 and are classified as not vulnerable to multidimensional poverty (MPI score: 0–0.19), vulnerable to multidimensional poverty (MPI score: 0.20–0.32), multidimensionally poor (MPI score: 0.33–0.49), and severely multidimensionally poor (MPI score: ≥0.50).

^c^Other regimens included abacavir + lamivudine + dolutegravir (n = 82), lamivudine + zidovudine + dolutegravir (n = 8), lamivudine/zidovudine 150 mg/300 mg tablet (n = 1), and abacavir + lamivudine + efavirenz (n = 1).

^d^Suppressed defined as <1000 copies/mL according to Ugandan guidelines; n = 2 missing.

### Adverse Event Frequency, Typology, and Timing

**Figure 1.**
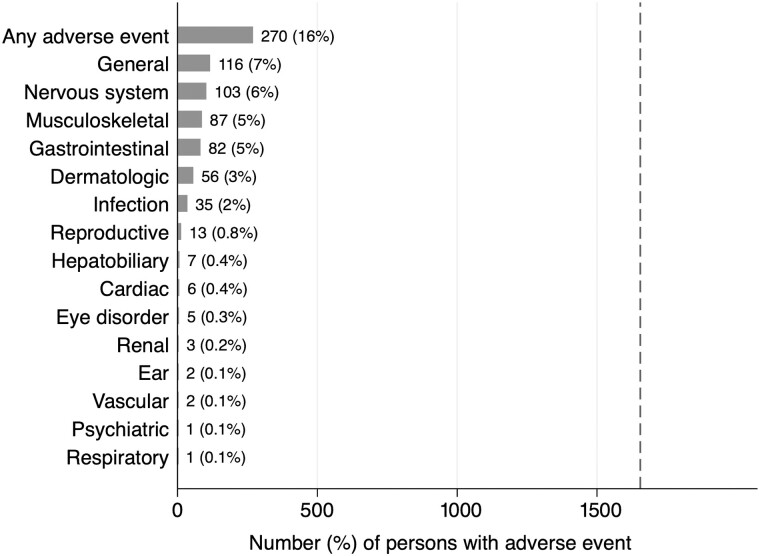
Frequency of reported adverse events (AEs) during isoniazid and rifapentine treatment for 12 weeks (3HP) according to Common Terminology Criteria for Adverse Events (version 5.0) categories. Shown are the number and percentage of participating people with human immunodeficiency virus taking 3HP for prevention of tuberculosis who reported AEs in each category. The total number of participants assessed for AEs in the 3HP Options Trial was 1655, as indicated by the dashed vertical line.

Overall, 270 (16.3%) participants reported at least 1 AE over the course of 3HP treatment (Figure 1), including 175 (19.0%) people receiving 3HP via DOT and 95 (12.9%) receiving 3HP via SAT (Table 1). Participants in the DOT arm, those who were female, or people with multidimensional poverty [12] were more likely to report any AE, associations confirmed in multivariable logistic regression models (adjusted odds ratio [aOR], 1.59 [95% confidence interval {CI}, 1.21–2.09], *P* = .001 for DOT; aOR, 1.61 [95% CI, 1.05–2.01], *P* = .02 for female sex; and aOR, 1.40 [95% CI, 1.00–1.96], *P* = .05 for poverty or severe multidimensional poverty) (Supplementary Table 1).

The most frequently reported AEs (reclassified according to CTCAE; Supplementary Table 2) were general disorders such as fever or flulike illness (n = 116 PWH [7% of all study participants]), nervous system disorders (n = 103 [6%]), musculoskeletal disorders (n = 87 [5%]), gastrointestinal disorders (n = 82 [5%]), or dermatologic disorders (n = 56 [3%]) (Figure 1). Hepatobiliary disorders were infrequent, with only 7 participants (0.4%) reporting related AEs.

**Figure 2.**
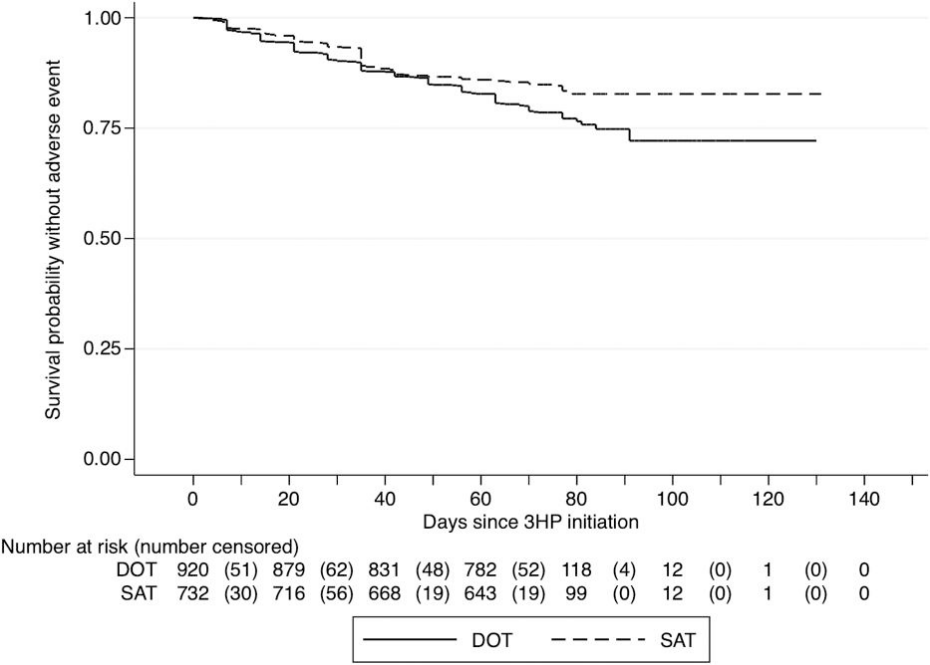
Time to first adverse event (AE) experience, stratified by isoniazid and rifapentine treatment for 12 weeks (3HP) delivery strategy. The solid horizontal stepped line represents the cumulative probability of not having an AE among participants receiving 3HP via facilitated directly observed therapy (DOT), and the dashed horizontal stepped line represents those receiving 3HP via facilitated self-administered therapy (SAT). The numbers outside of parentheses at the bottom of the figure represent the total number of participants at risk, while the numbers in parentheses represent the number censored at the time interval indicated.

Among participants who had an AE, the median time to an AE was 35 days (IQR, 14–49 days). There was a significant difference in the survival curves comparing time to an AE between participants receiving 3HP via DOT versus SAT, with people receiving 3HP by SAT less likely to report an AE (log-rank *P* = .001; Figure 2).

### Additional Healthcare Utilization for Adverse Events

Among those reporting AEs, 211 (78.1%) were referred to a clinician for further evaluation, 202 (74.8%) required laboratory testing, and 102 (37.8%) had medication(s) prescribed. The most common types of laboratory tests ordered included urinalysis (n = 77), malaria tests (n = 59), and blood glucose tests (n = 24). For the 209 total laboratory tests ordered, 35 diagnoses were made (Supplementary Figure 1). The proportion referred to a clinician was similar among those taking 3HP via facilitated DOT versus facilitated SAT (*P* = .22). Those who took 3HP by facilitated SAT were more likely to have laboratory testing ordered (46.3% vs 33.1%, *P* = .04), whereas those who took 3HP by facilitated DOT were more likely to be prescribed medications to treat AEs (78.9% vs 67.4%, *P* = .04).

Among the 270 PWH reporting AEs, 40 (14.8%) had treatment held temporarily and 14 (5.1%) had treatment permanently discontinued by a Mulago ISS clinician (<1% of PWH taking 3HP overall). Participants taking 3HP via SAT (28.4% vs 15.4%) and participants enrolled after the first 6 months of the study (22.1% vs 15.7%) were more likely to have 3HP treatment held or discontinued due to an AE (Supplementary Table 3).

## DISCUSSION

Growing interest in the use of short-course TPT regimens such as 3HP for TB prevention requires better data on AEs to inform planning for programmatic scale-up. In this pragmatic trial of 1655 PWH taking 3HP in Kampala, Uganda, with minimal loss to follow-up prior to treatment completion, the frequency of AEs was low, with only 16% of participants experiencing any AE. Adverse events were effectively managed by routine healthcare providers, with no deaths, only 3% requiring treatment interruption, and <1% requiring treatment discontinuation. These data add to the existing body of literature demonstrating 3HP safety and tolerability as some of the first data to come from a high-burden, programmatic context, and may provide reassurance to programs planning for or considering short-course regimens as alternatives to TPT in similar settings.

The frequency of AEs reported in this pragmatic trial was comparable to what has been documented in other recent studies of 3HP implementation in high-burden settings [13, 14] but substantially lower than what was reported from a multicountry randomized trial of 3HP [15], in which 77% reported 1 or more symptoms at any time during treatment [11]. However, data from the only participating African country in that trial included only a small number of participants (n = 83). Additionally, treatment dosing/supervision, study follow-up, and AE management in that trial were managed by trained research staff. Here, we provide data from people taking 3HP in the context of routine HIV/AIDS care, which may be a more accurate reflection of what can be expected in other programmatic settings. In this context, although the majority of participants who reported an AE were prescribed medications for those symptoms, the burden of laboratory testing was relatively modest.

As expected, we found that AEs were more commonly reported with DOT than with SAT. DOT provides more opportunities for health workers to directly ask about and screen for potential side effects. It is also possible that PWH had a lower threshold for reporting potential side effects when face-to-face with a health worker than when prompted to do so via IVR phone calls. Given the similar treatment completion rates between DOT and SAT, it is unlikely serious AEs were more likely to be reported with one versus the other.

We found that being female and experiencing multidimensional poverty were associated with a higher likelihood of reporting AEs. The association between female sex and AEs while on 3HP treatment is not fully understood, but it has been noted in previous studies on 3HP [11, 14, 16, 17]. Poverty is a well-documented social determinant of negative TB and TPT outcomes, particularly due to its links to undernutrition, poor health knowledge, and a lack of empowerment to utilize that knowledge [18–20].

A strength of this study was its pragmatic nature, including management of AEs by routine healthcare providers. We therefore provide some of the first real-world evidence of 3HP AE occurrence from a clinical setting in a high-burden country. Limitations include the lack of a formal cost analysis of AE management and the possibility that people included in our study were more carefully screened for AEs than would typically occur in the absence of a research study. As such, while we may overestimate the frequency of AEs, management of those events, including decisions to withhold or discontinue 3HP, may have been more careful.

## CONCLUSIONS

This analysis of a pragmatic implementation trial in a large HIV clinic in Kampala, Uganda, reaffirms the safety of 3HP, with only 1 in 6 participants reporting an AE and <1% requiring permanent treatment discontinuation. These data also confirm that routine clinical staff can effectively manage 3HP-related AEs. These data can be useful to national TB programs and other implementing partners as they plan resources to support broader roll-out of short-course TPT, including an understanding of the potential additional time required and/or training needed for healthcare staff to manage 3HP-related AEs and the potential additional costs for anticipated laboratory tests ordered and medications prescribed associated with 3HP-related AEs. Based on our findings, these additional resources and/or costs associated with 3HP-related AEs are likely to be low. Future studies of the total cost of 3HP-related AE management could provide a more complete picture of the AE profile likely to be experienced during programmatic scale-up. Nonetheless, these pragmatic data indicate that 3HP can be delivered safely and effectively in routine settings and can help implementers plan resources for AE management.

## Supplementary Data

Supplementary materials are available at *Open Forum Infectious Diseases* online. Consisting of data provided by the authors to benefit the reader, the posted materials are not copyedited and are the sole responsibility of the authors, so questions or comments should be addressed to the corresponding author.

## Supplementary Material

ofae667_Supplementary_Data

## Notes

***Author contributions*.** J. L. K.: Formal analysis, data curation, writing–original draft, writing–review and editing, visualization, project administration. A. M.: Data curation, validation, data curation, writing–review and editing, project administration. H. E. A.: Formal analysis, data curation, writing–original draft, writing–review and editing. L. A., F. W., and J. N.: Data curation, writing–review and editing. C. A. B.: Project administration, writing–review and editing. N. K.: Conceptualization, methodology, supervision, writing–review and editing. P. P. J. P: Formal analysis, writing–review and editing. A. K.: Conceptualization, methodology, investigation, supervision, writing–review and editing. D. W. D.: Conceptualization, methodology, investigation, supervision, project administration, writing–review and editing. A. C.: Conceptualization, methodology, investigation, supervision, project administration, funding acquisition, writing–review and editing. F. C. S.: Conceptualization, methodology, investigation, supervision, project administration, resources, writing–review and editing.

***Acknowledgments*.** The authors thank the administration, staff, and patients at the Mulago ISS clinic for their time and participation, and the research administration of the Infectious Diseases Research Collaboration and Walimu.

***Data availability*.** Data are not publicly available.

***Financial support*.** This work was supported by the National Heart, Lung, and Blood Institute of the US National Institutes of Health (grant number R01HL144406 to A. C.).
